# Inhibition of hypoxia inducible factor-1α attenuates abdominal aortic aneurysm progression through the down-regulation of matrix metalloproteinases

**DOI:** 10.1038/srep28612

**Published:** 2016-07-01

**Authors:** Shih-Hung Tsai, Po-Hsun Huang, Yu-Juei Hsu, Yi-Jen Peng, Chien-Hsing Lee, Jen-Chun Wang, Jaw-Wen Chen, Shing-Jong Lin

**Affiliations:** 1 Department of Emergency Medicine, Tri-Service General Hospital, National Defense Medical Center, Taipei, Taiwan; 2 Institute of Clinical Medicine, National Yang-Ming University, Taipei, Taiwan; 3 Division of Cardiology, Department of Internal Medicine, Taipei Veterans General Hospital, Taipei, Taiwan; 4 Cardiovascular Research Center, National Yang-Ming University, Taipei, Taiwan; 5 Division of Nephrology, Department of medicine, Tri-Service General Hospital, National Defense Medical Center, Taipei, Taiwan; 6 Department of Pathology, Tri-Service General Hospital, National Defense Medical Center, Taipei, Taiwan; 7 Division of Endocrinology, Department of medicine, Tri-Service General Hospital, National Defense Medical Center, Taipei, Taiwan; 8 Institute and Department of Pharmacology, National Yang-Ming University, Taipei, Taiwan; 9 Department of Medical Research and Education, Taipei Veterans General Hospital, Taipei, Taiwan

## Abstract

Hypoxia inducible factor-1α (HIF-1α) pathway is associated with many vascular diseases, including atherosclerosis, arterial aneurysms, pulmonary hypertension and chronic venous diseases. Significant HIF-1α expression could be found at the rupture edge at human abdominal aortic aneurysm (AAA) tissues. While our initial *in vitro* experiments had shown that deferoxamine (DFO) could attenuate angiotensin II (AngII) induced endothelial activations; we unexpectedly found that DFO augmented the severity of AngII-induced AAA, at least partly through increased accumulation of HIF-1α. The findings promoted us to test whether aneurysmal prone factors could up-regulate the expression of MMP-2 and MMP-9 through aberrantly increased HIF-1α and promote AAA development. AngII induced AAA in hyperlipidemic mice model was used. DFO, as a prolyl hydroxylase inhibitor, stabilized HIF-1α and augmented MMPs activities. Aneurysmal-prone factors induced HIF-1α can cause overexpression of MMP-2 and MMP-9 and promote aneurysmal progression. Pharmacological HIF-1α inhibitors, digoxin and 2-ME could ameliorate AngII induced AAA *in vivo*. HIF-1α is pivotal for the development of AAA. Our study provides a rationale for using HIF-1α inhibitors as an adjunctive medical therapy in addition to current cardiovascular risk-reducing regimens.

Abdominal aortic aneurysm (AAA) rupture can be life threatening and is a common cause of sudden death among the elderly^1^,^2^. Unfortunately, AAA mortality is not declining globally^3^. Up to 12.5% of men over 75 years of age have an aortic aneurysm^1^. The risk of AAA rupture is determined by its size: rupture occurs in approximately 2% of AAAs less than 4 cm in diameter and in more than 25% of AAAs larger than 5 cm. Surgical repair is advised for large AAAs (diameter >5.5 cm) and that have a growth rate in excess of 1 cm/year^4^,^5^. Most aortic aneurysms are detected incidentally, and 90% of these aneurysms are below the threshold for intervention at the time of detection. Thus, developing effective medical treatments that inhibit AAA expansion could change the current approach to aneurysm management^6^ The pathogenesis of AAA is characterized by the degradation of the extracellular matrix (ECM) by the increased generation of Reactive oxygen species (ROS), matrix metalloproteinase (MMPs), and inflammatory reactions^7^. Several studies suggest that endothelial activation and dysfunction are also pivotal in the pathogenesis of aortic aneurysms and in AngII mediated aortic dissections^8^,^9^,^10^,^11^,^12^. Although medications to reduce vascular inflammation and inhibit MMPs have been proposed to treat growing AAA, recent clinical trials have found that mast cell stabilizers and doxycycline failed to reduce aneurysm growth and had no impact on AAA repair management^13^,^14^. Thus, in addition to the current treatments for reducing cardiovascular risk, an adjunctive medical therapy targeting the regulation of ECM metabolism is still required.

The protein hypoxia inducible factor-1α (HIF-1α) accumulates in the cytoplasm under hypoxic conditions and translocates to the nucleus to heterodimerize with HIF-1b, forming an active transcription factor. HIF-1α has been implicated in the pathogenesis of atherosclerosis, AAA formation and pulmonary hypertension^15^. HIF-1α overexpression could be found at the rupture edge at human AAA tissues^16^. On the other hand, iron chelation has been shown to stabilize HIF-1α by inhibiting the HIF-1α degradation enzyme prolyl hydroxylase (PHD). However, researches regarding the roles of HIF-1α in the pathogenesis of AAA are still limited. Iron is involved in the pathogenesis of AAA with oxidative stress and inflammation, both in human AAA tissues and in AngII-induced AAA in ApoE^−/−^ mice^17^. As an iron chelator, deferoxamine (DFO) could ameliorate oxidative stress, inflammatory cytokines productions, macrophage infiltration, NF-kB activation and adhesion molecule expression in several animal models, including in treating atherosclerotic vascular diseases by reducing oxidative stress and inflammation^18^,^19^,^20^,^21^. While our initial *in vitro* experiments showed that DFO attenuated AngII-induced endothelial dysfunction and activation, we unexpectedly found that DFO augmented the severity of AngII-induced AAA, partially due to an aberrant increased HIF-1α, MMP-2 and MMP-9 expression. The present study aimed to test whether aneurysmal-prone factors could up-regulate the expression of MMP-2 and MMP-9 through aberrantly increased HIF-1α and further promote the development and progression of AAA. We also provide a rationale for using pharmacological HIF-1α inhibitors as an adjunctive medical therapy for AAA.

## Materials and Methods

### Cell cultures and reagents

Human aortic endothelial cells (HAECs) used in experiments testing the effects of DFO on vascular cell biology were purchased from the Life Technologies. Angiotensin II (Ang II) and nicotine were purchased from Sigma-Aldrich. Oxidized-1-palmitoyl-2-arachidonyl-sn-glycerol-3-phosphocholine (oxPAPC), 2-methoxyestradiol (2-ME) and digoxin were purchased from invivoGen, Abmole and GSK respectively.

### Preparation of HIF-1α plasmid and transfection of short hairpin RNA

A human HIF-1α open reading fragment was obtained from the Mammalian Gene Collection and reconstructed into a pOTB7 plasmid vector. The insertion in the new plasmid (pOTB7-HIF-1α) was confirmed using DNA sequencing. The pOTB7-HIF-1α and empty plasmids were purified using Midi Plasmid Kit PI025 (Geneaid). The purity of the plasmids was verified with the absorbance ratio at 260 and 280 nm and by 1% agarose gel electrophoresis. Short hairpin RNAs (shRNAs) plasmids to knockdown HIF-1α and scrambled control were provided by National RNAi Core Facility of Academia Sinica, Taipei, Taiwan. Transfection was performed using the Lipofectamine^®^ 3000 (ThermoFisher Scientific), as the recommendation of the manufacturer.

### Immunoblotting

After the careful removal of peri-aortic soft tissue, the whole aorta was saline-perfused and excised. The aorta was homogenated, and protein lysates were subjected to SDS-PAGE followed by transfer onto a PVDF membrane. Membranes were probed with monoclonal antibodies against p-JNK (CST, #9251), JNK (CST, #9252), p-ERK (CST, #9106), ERK (CST, #4695), p-P65 (CST, #3033), VEGF (BD Biosciences, 555036), intercellular adhesion molecule (ICAM, Santa Cruz, SC-1511), vascular cell adhesion molecule (VCAM, Santa Crus, SC-1504), HIF-1α (GeneTex, GTX127309), total eNOS (CST, #9586) and phosphorylated eNOS (p-eNOS, #9574), Kruppel-like factor (KLF4, CST, #4038), SIRT1-mouse specific (CTS, #3931), SIRT1-human specific (CST, #2496), MMP-2 (CST, #4022), MMP-9 (CST, #G657) and β-actin. Bands were visualized by chemiluminescence detection reagents. Densitometric analysis was conducted with imaging processing software (Multi Gauge, Fujifilm), and data were expressed as a fold change relative to the controls.

### Measurement of ROS production

The homogenates of the cell lysates were stained with 2′,7′-Dichlorofluorescin diacetate (DCFH-DA). DCFH-DA was oxidized by ROS to form the highly fluorescent 2′,7′-dichlorofluorescein. The samples were loaded onto 96-well plates for 30 minutes at 37 °C, and fluorescence intensity was measured with an excitation of 488 nm and an emission of 520 nm.

### Measurement of the activities of matrix metalloproteinases

Gelatin zymography was used to determine the gelatinolytic activities of the MMP-2 and MMP-9 activities of the homogenates of the aorta and conditioned medium as previously described. In brief, equivalent amount of samples were electrophoresed under non-reducing conditions onto 7.5% SDS polyacrylamide gels containing 0.1 mg/ml gelatin as substrate. The gels were washed in a buffer containing 2.5% Triton X-100 for one hour to remove SDS and incubated with a substrate buffer at 37 °C for 18 hours. The MMP activities were then quantified by densitometry scanning.

### Chromatin immune-precipitation assay

Chromatin immunoprecipitation (CHIP) assays were performed as previously described^22^. In brief, confluent cells were cross-linked with 4% PFA and then ceased by adding glycerin. Cells were then washed with cold PBS and collected using a FA lysis buffer. After shearing with sonication, the HIF-1α-bound chromatin was immunoprecipitated by rabbit anti-HIF-1α (GeneTex, GTX127309) and mouse IgG (Cell Signaling) linked to protein A/G Dynabeads (Invitrogene). Protein and RNA were then degraded by Proteinase K (100 μg) and RNase A (1 μg), respectively. The purified chromatin DNA was subjected to real time-quantitative PCR.

### Primer Sequences Used in Chromatin immune-precipitation assay

To predict potential HIF-1 binding sites, hypoxia response element (HRE) on selected human and mouse genes was analyzed using the position weight matrix algorithm from TRANSFAC15 to scan the promoter regions of each gene. The promoter region was defined as −5000 to +5000 nucleotides from the transcriptional start site. The sequences of the primers used to detect HIF-1α binding to the three HIF-1α binding sites (HRE1, HRE2) in the human MMP-2 and MMP-9 promoter regions are shown in Supplementary Table 4.

### Angiotensin II induced abdominal aortic aneurysm model

This is a prospective interventional animal study. Male low-density lipoprotein receptor (LDLR^−/−^) mice on a C57BL/6J background were obtained from Jackson Lab. Mice were fed with high fat diet and kept *ad libertum*. Alzet osmotic minipumps (model 2004; ALZET Scientific Products, Mountain View, California, USA) were implanted into mice at 8–10 weeks of age. Pumps filled with solutions of AngII (Sigma Chemical CO., St. Louis, Missouri, USA) delivered 1000 ng/kg/min of AngII for 28 days as previously described^23^. The pumps were placed into the subcutaneous space of mice through a small incision in the back and were then closed with surgical clips. Avertin (tribromoethanol, 250 mg/kg, intraperitoneally) was used for anaesthesia and mice were considered as adequately anaesthetized when no attempt to withdraw the limb after pressure could be observed. DFO was given 100 mg/kg/day intraperitoneally as previously described in the treatment of atherosclerosis. Digoxin (1.25 mg/kg/day) was given subcutaneously as previously described in inhibiting HIF-1 synthesis and HIF-1α transcriptional activities with the serum concentration of digoxin in at or below the therapeutic range for humans (0.5–2.0 ng/ml)^24^,^25^,^26^,^27^,^28^. 2-methoxyestradiol (50 mg/kg/day) was given subcutaneously. The animals were monitored during the treatment for their body weight to assess side-effects. At the end of the study, mice were euthanized by exsanguination under anaesthesia. Blood was withdrawn from the right heart ventricle for analysis. All experimental protocols and procedures were approved by the institutional animal care committee of the National Defense Medical Center (Taipei, Taiwan) and complied with the Guide for the Care and Use of Laboratory Animals published by the US National Institutes of Health (8^th^ edition, 2011)^29^.

### Determination of blood pressure and serum levels of lipid profiles

Systolic blood pressure and lipid profiles were determined weekly. Systolic blood pressure was measured in conscious mice using tail cuff apparatus (Softron BP-98A tail blood pressure system, Tokyo, Japan). Serum was obtained by centrifugation at 3,000 rpm for 10 minutes at room temperature. Serum total cholesterol, triglyceride, HDL-cholesterol, LDL-cholesterol, total calcium and creatinine levels were determined enzymatically (Arkray Kyoto, Japan).

### Characteristics and quantification of AAA

After perfusion and fixation with cold 4% paraformaldehyde, the aorta was exposed under a dissecting microscope, and the periadventitial tissue was removed from the aortic wall. The morphology of the aorta was photographed *in situ*, and the maximal external diameter of the suprarenal aorta was measured using imaging processing software (Image J). A definition of outgrowth of more than 50% indicated the development of aortic aneurysm as previously described. The characteristics of an AAA were scored as following as previously described: Type I represents a small single dilation (1.5–2.0 times of a normal diameter); Type II denotes a large single dilation (>2 times of a normal diameter); Type III is multiple dilations; and Type IV is an aortic rupture that lead to death due to bleeding into the peritoneal cavity^30^.

### Histology and Immunohistochemistry

Perfusion-fixed aortas were embedded, cut in cross section (5 μm) and stained with hematoxylin and eosin. Verhoeff-Van Geisen (VVG) was used for detecting elastin. The severity of elastin degradation was semi-quantified as previously described^31^. Immunohistochemistry staining of HIF-1α was used to reveal the aberrantly increased HIF-α in the aorta.

### Statistical analysis

All experiments were performed at least three times. All continuous variables were presented as the mean ± standard error of the mean (SEM). Prior to statistical analysis data was tested for equality of variance by Bartlett’s test. Statistical significance was evaluated using the unpaired Student t-test for comparisons between 2 means. Two-sample nonparametric comparisons were performed using a Chi-square test. Comparisons between multiple groups were analyzed by one-way analysis of variance to assess the significance (ANOVA) and *post-hoc* analysis was performed using the Bonferroni test. Statistical significance was defined as a p-value of less than 0.05. Analyses were performed using a statistical software package (Prism version 5, GraphPad Software, La Jolla California USA). All images shown are examples of replicates and are representative for the respective groups.

## Results

### DFO attenuates AngII-induced endothelial cell activation and dysfunction *in vitro* but does not protect against AngII-induced AAA

The detrimental effects of AngII on accelerated atherosclerosis and aneurysmal formation have been attributed to MAPK JNK and ERK activations^9^,^11^. We found that DFO attenuated AngII-induced JNK, ERK1/2 and ROS production. In addition, DFO dose-dependently attenuated AngII-induced down-regulation of the atheroprotective factors eNOS, SIRT1 and KLR4 *in vitro* (n = 4–5, Supplementary Figure 1). Despite the promising *in vitro* effects, DFO unexpectedly had the trend toward increasing the incidence of AngII-induced AAA (0% vs. 75% vs. 85%, p = 0.69) and the external diameter of the aorta (0.67 ± 0.11 vs. 1.62 ± 0.83 vs. 1.98 ± 1.07 mm, p-values: saline vs. AngII, p < 0.05; Saline vs. AngII + DFO, p < 0.05; and AngII vs. AngII + DFO, n.s., Supplementary Figure 2A,B). In addition, we observed a significant increase in the incidence of type III (multiple aneurysms) and type IV (rupture) AAA in DFO treated mice (35% vs. 70% p < 0.05, n = 20 in each group, Supplementary Figure 2C). DFO did not alter systolic blood pressure (SBP), serum cholesterol and creatinine. The dose of DFO used in the present study did not cause significant weight loss (less than 10% of body weight) or changes in BP during the study period.

### Increased HIF-1α expression was associated with increased MMP-2 and MMP-9 activity *in vitro*

It is known that DFO could also cause HIF-1α accumulation by inhibiting PHD^32^. The unexpected findings of DFO aggravated the severity of AngII induced AAA, independent of its ability in preventing AngII induced ECs activation had led us to explore the roles of HIF-1α in the pathogenesis of AAA. To further elucidate how DFO treatment could augment AAA severity, we tested HIF-1α expression in the whole aorta lysate and found that compared with the AngII-treated group, DFO treated mice had significantly higher HIF-1α expression in the aorta lysate and significantly increased MMP-2 and MMP-9 activities (n = 5, Supplementary Figure 3).

### Aberrantly induced HIF-1α expression directly up-regulated MMP-2 and MMP-9

**Figure 1:**
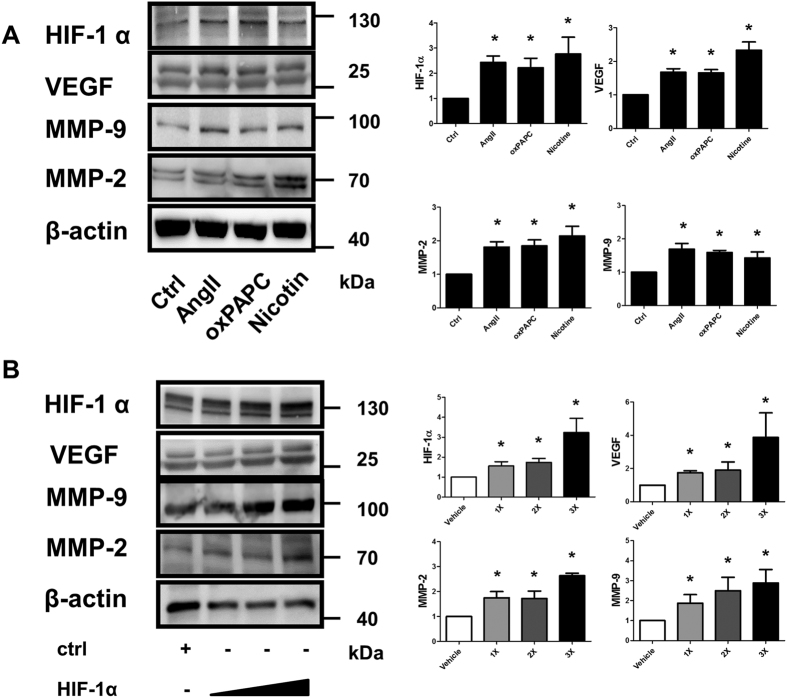
Increased HIF-1α expression was associated with increased MMP-2 and MMP-9 activity *in vitro.* (**A**) Several aneurysmal prone factors, including AngII, oxPAPC and nicotine, upregulated the expression of HIF-1α and MMP-2 and MMP-9 expression concurrently. (**B**) Overexpression of HIF-1α dose-dependently upregulated the expression of MMP-2 and MMP-9. The gels had been run under the same experimental conditions. n = 4 to 5 per group. *P < 0.05 vs control.

**Figure 2:**
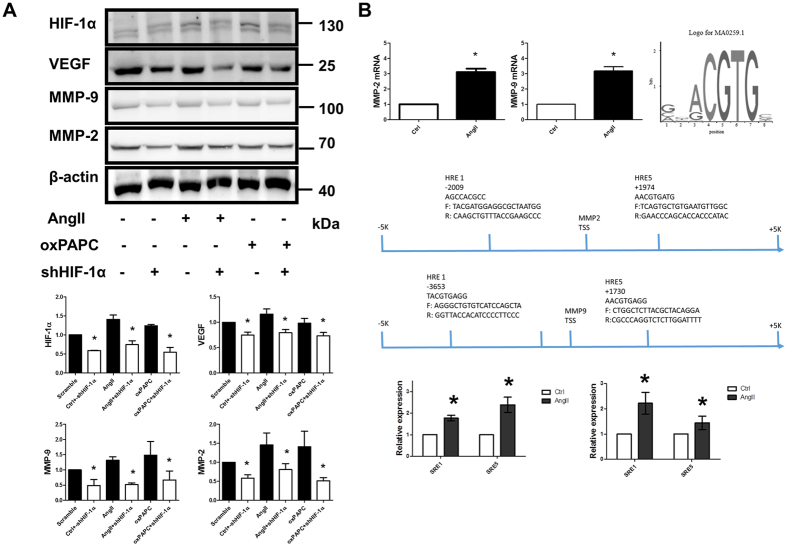
Aberrantly induced HIF-1α expression directly up-regulated MMP-2 and MMP-9. (**A**) Silencing HIF-1α could also ameliorate AngII and oxPAPC induced by MMP-2 and MMP-9 expression. (**B**) As expected, AngII could induce MMP-2 and MMP-9 overexpression. Bioinformatics prediction models revealed the presence of several conservative HREs in the promotor regions of MMP-2 and MMP-9. A CHIP assay confirmed that AngII aberrantly up-regulated MMP-2 and MMP-9 through HIF-1α. The gels have been run under the same experimental conditions. n = 5 per group. *P < 0.05.

Poorly-controlled hypertension and hyperlipidemia and a high prevalence of smoking were associated with persistent AAA-related mortality^3^. In the AngII induced AAA in hyperlipidemic mice model, increased levels of cholesterol and AngII promoted arterial inflammation and set the trajectory for the size and evolution of AAA^33^. To further decipher the association between the aberrantly increased HIF-1α and MMPs, three aneurysmal prone factors, AngII, oxPAPC and nicotine, were tested in HAECs. We found that these aneurysm prone factors significantly and concurrently induced HIF-1α, MMP-2 and MMP-9 expression (n = 4–5, Fig. 1A). Overexpression of HIF-1α dose-dependently up-regulated the expression of MMP-2 and MMP-9 (n = 4–5, Fig. 1B). Silencing HIF-1α also attenuated AngII- and Oxidized-1-palmitoyl-2-arachidonyl-sn-glycerol-3-phosphocholine (Ox-PAPC) -induced overexpression of MMP-2 and MMP-9 (n = 4–5, Fig. 2A). As expected, AngII could induce MMP-2 and MMP-9 overexpression. Bioinformatics prediction models revealed the presence of several conservative HREs in the promotor regions of MMP-2 and MMP-9 (Supplementary Table 1). By using a CHIP assay, we further confirmed that AngII could up-regulate MMP-2 and MMP-9 through aberrantly induced HIF-1α expression (n = 5, Fig. 2B).

### Both clinical relevant non-selective pharmacological HIF-1α inhibitors, 2-methoxyestradiol and digoxin, attenuate AngII- and oxPAPC-induced MMP-2 and MMP-9 expressions *in vivo* and AngII-induced AAA formation *in vivo*

**Figure 3:**
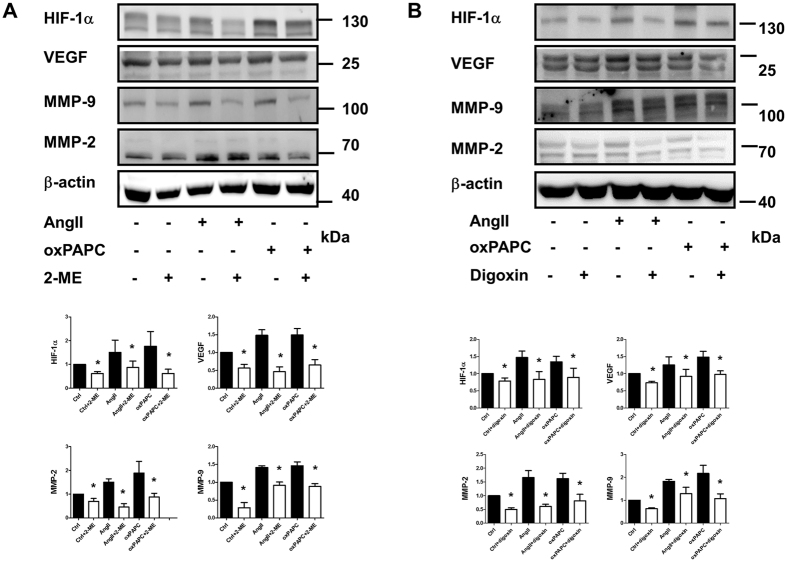
Pharmacological HIF-1α inhibitors attenuated AngII- and oxPAPC-induced MMP-2 and MMP-9 expression. Pharmacological inhibitors (**A**) 2-methoxyestradiol (2-ME, 6 μM) and **(B**) digoxin (100 nM) ameliorated AngII- and oxPAPC-induced upregulation of MMP-2 and MMP-9. We found that both HIF-1α inhibitors ameliorated AngII- and oxPAPC-induced upregulation of MMP-2 and MMP-9. The gels have been run under the same experimental conditions. n = 5 per group. *P < 0.05.

**Figure 4:**
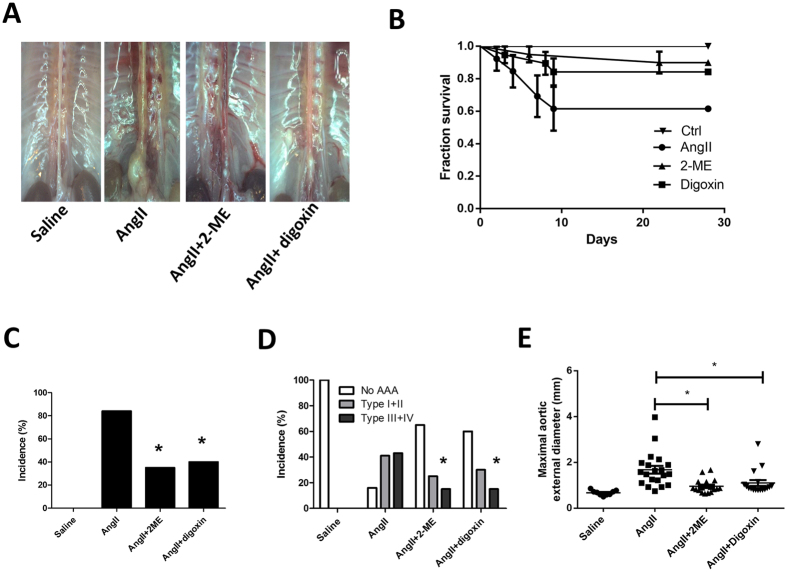
Pharmacological HIF-1α inhibitors digoxin and 2-methoxyestradiol (2ME) attenuated angiotensin II-induced abdominal aortic aneurysm *in vivo*. (**A**) Representative figures of aorta obtained from saline, AngII (1000 ng/kg/min), AngII + 2-ME (25 mg/kg/day, s.c.) and AngII + digoxin (1.25 mg/kg/day, i.p.) treated LDL^−/−^ mice. (**B**) Both 2-ME and digoxin significantly improved survival rates; (**C**) decreased the incidence of AAA (0% vs. 84% vs. 35%, vs. 40%, p < 0.01); as well the severity of AAA (0% vs. 45% vs. 15%, vs. 15%, p < 0.05); (**D,E**) the external diameter of the aorta (0.67 ± 0.11 mm vs. 1.68. ± 0.77 mm vs.0.96 ± 0.30 mm, vs. 1.10 ± 0.53 mm, p < 0.05. n = 20 per group. *P < 0.05.

**Figure 5:**
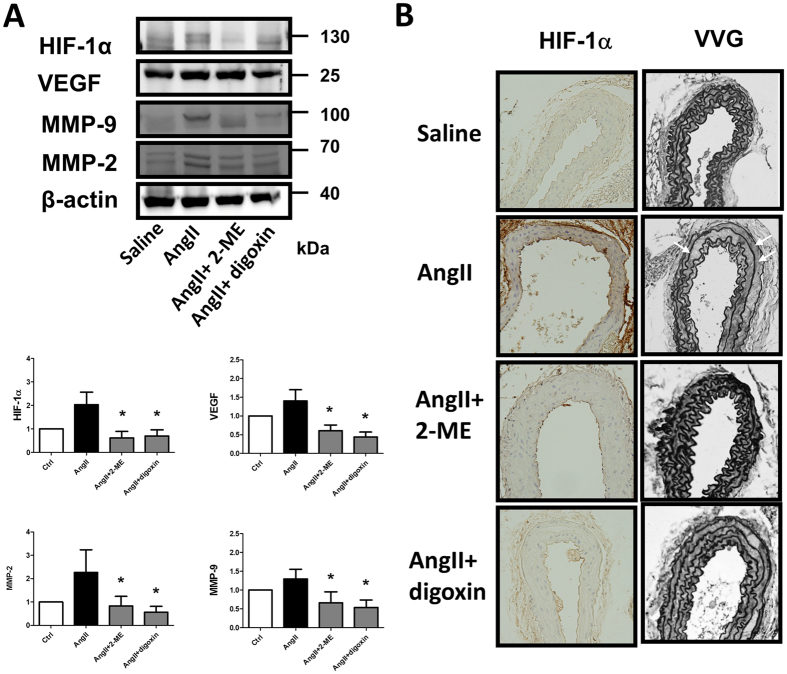
Pharmacological HIF-1α inhibitors 2-methoxyestradiol and digoxin decreased matrix metalloproteinase expression and alleviated angiotensin II-induced elastin degradation *in vivo*. (**A**) Administration of 2-ME and digoxin significantly decreased AngII- mediated HIF-1α, VEGF, MMP-2 and MMP-9 overexpression in the homogenates of aortas. (**B**) Immunohistochemistry staining for HIF-1α and VVG staining revealed reduced HIF-1α expression and decreased elastin degradation. The gels have been run under the same experimental conditions. n = 4–6 per group. *p < 0.05. compared to AngII.

We then tested whether the pharmacological HIF-1α inhibitors 2-methoxyestradiol (2-ME, 6 μM) and digoxin (100 nM) could attenuate AngII- and oxPAPC-induced upregulation of MMP-2 and MMP-9. We found that both 2-ME and digoxin attenuated AngII and oxPAPC induced up-regulation of MMP-2 and MMP-9 (n = 5, Fig. 3). To further provide a rationale for using HIF-1α inhibitors as an adjunctive medical therapy for small AAAs, we tested whether pharmacological HIF-1α inhibitors could attenuate AngII-induced AAA *in vivo*. As shown in Fig. 4 and the representative figures of aorta of each groups (Fig. 4A), both non-selective HIF-1α inhibitors 2-ME (25 mg/kg/day, s.c.) and digoxin (1.25 mg/kg/day, i.p.) significantly improved the survival rates (log rank test, n = 20 in each group, p < 0.05, Fig. 4B); decreased the incidences of AAA (0% vs. 84% vs. 35%, vs. 40%, p < 0.01, Fig. 4C), the severities of AAA, in terms of less type III and type IV lesions (43% vs. 15%, vs. 15%, p < 0.05, Fig. 4D) and the external diameters of the aorta (0.67 ± 0.11 mm vs. 1.68. ± 0.77 vs. 0.96 ± 0.30 vs. 1.10 ± 0.53 mm, p < 0.05, Fig. 4E). Administration of 2-ME and digoxin significantly decreased AngII- mediated HIF-1α, VEGF, MMP-2 and MMP-9 overexpression in the homogenates of the whole aortas, as shown in Fig. 5A. 2-ME and digoxin decreased the AngII induced HIF-1α levels accumulation and elastin degradation in the aorta (Fig. 5B). The dose of 2-ME and digoxin used in present study did not alter SBP, serum cholesterol or creatinine levels or cause significant weight loss (less than 10% of body weight) during the study period, which is consistent with previous reports^27^,^34^.

## Discussion

In the present study, we are able to show the pivotal roles of HIF-1α in the pathogenesis of AAA by using the combination of three unspecific clinical-relevant HIF-1α modulators, i.e., DFO, 2-ME and digoxin. Although DFO could attenuate AngII-induced endothelial activation, i.e. the reduction of ROS production as well reduction of MAPK JNK and ERK1/2 activations *in vivo* and *in vitro*. DFO, also as a PHD inhibitor, stabilized HIF-1α and augmented MMPs activities. As a result, the promising anti-inflammatory effects could not confer protection against AngII-induced AAA *in vivo*. The findings promoted us to decipher the roles of aberrantly induced HIF-1α in the progression of AAA. We found that several aneurysmal prone factors can aberrantly induce HIF-1α, leading to the overexpression of MMP-2 and MMP-9, and can promote aneurysmal progression. We further confirmed that two pharmacological HIF-1α inhibitors, digoxin and 2-ME, attenuated AngII-induced AAA, providing a rationale for using HIF-1α inhibitors as an adjunctive medical approach for small AAA.

HIF-1α is the master transcriptional factor for the adaption to hypoxia in vertebrate animals. HIF-1α can upregulate MMP-2 and MMP-9 in many cell types^35^,^36^,^37^,^38^. A positive correlation exists between global trends in systolic blood pressure, cholesterol, smoking prevalences and AAA related mortalities^3^. In the present study, we showed that several representative aneurysmal-prone factors may lead to HIF-1α accumulation. AngII can stimulate HIF-1α accumulation by increasing HIF-1α transcription and by inhibiting PHD activity, which would also lead to HIF-1α accumulation^39^. Chronic infusion of AngII increased HIF-1α levels in the kidney, and hyper-activation of HIF-1α in the kidney plays a crucial role in AngII-mediated chronic kidney injury^40^. In VSMC and macrophages, HIF-1α promotes vascular inflammation and vascular remodeling^41^,^42^. OxPAPC is reported to induce inflammatory gene expression and stimulate JNK and p38 signaling in a toll-like receptor (TLR) 2-dependent manner^43^. TLR4 activation is necessary for an augmented pulmonary HIF-1α response in hemorrhagic shock-induced lung injury^44^. Nicotine could induce the activation of HIF-1α and VEGF through α5 and α7 nicotinic acetylcholine receptors^45^,^46^. In the current study, we showed that these aneurysmal-prone factors promoted aneurysm progression at least partly through aberrantly increased HIF-1α expression.

Although AAA had previously been described as a form of atherosclerosis, it is now recognized as a distinct process involving all of the layers of the vessel wall. Several cellular types, including VSMCs, macrophages, neutrophils, T cells, mast cells and peri-adventitial fibroblasts have been shown to involve in the pathogenesis of AAA^34^,^47^,^48^,^49^. However, emerging evidences suggest the pivotal roles of ECs during the progression of AAA by initiating vascular inflammation, expressing adhesion molecules and activating inflammatory cascades, which may further trigger macrophage infiltration and inflammation in the adventitia and media^9^,^10^. Loss of the endothelium and its replacement by a thick thrombus are also structural features of human AAAs^48^,^50^,^51^. Previous studies suggest that AAA could be attenuated by the administration of drugs that reduce endothelial activation and restoring the endothelial lining were efficient therapy against AAA expansion^9^,^11^,^50^,^51^. AngII infusion could induce endothelial miR-712/miR-205 in AAA, both *in vitro* and *in vivo*, and the *s*ilencing of miR-712/miR-205 by antisense oligonucleotides could decrease inflammation and the activity of endothelail MMPs, thus preventing AAA development in AngII-infused ApoE^−/−^ mice^52^. Exogenous VEGF could further enhace AngII induced AAA in ApoE^−/−^ mice provide some clues for the involvement of ECs in the progression of AAA^53^. Taken together, our current study further revealed that those aneurysmal-prone factors could cause MMP-2 and MMP-9 overexpression through aberrantly increased HIF-1α in the ECs.

Oxidative stress is induced early during the course of AAA formation, and a growing body of evidence suggests that nicotinamide adenine dinucleotide phosphate (NADPH) oxidase is a source of oxidative stress in the pathogenesis of AAA. AngII can increase ROS production via NADPH and further increase adhesion molecule expression and inflammatory cell recruitment and further activate stress signals^54^. Consistent with our findings in ECs, Kazi., *et al*. had found that DFO could ameliorate AngII induced JNK activation in THP-1 cells^55^. Although iron restriction diet had been shown to ameliorate AngII-induced AAA in ApoE^−/−^ mice, the *in vivo* effects of DFO on AAA progression had not been further tested^55^. On contrary to previous studies showing that DFO inhibits inflammation and atherosclerosis in ApoE^−/−^ mice and cholesterol-fed rabbits^19^, the current study demonstrated that DFO potentiated AngII-induced AAA in hyperlipidemic mice through the accumulation of HIF-1α. We highlight that the potential beneficial iron depletion effects could be potentially overwhelmed by the increased HIF-1α accumulation.

Anti-inflammatory therapies using mast cell stabilizers and doxycycline failed to limit the expansion of preexisting AAAs^13^,^14^. In addition, the enlargement of preexisting AAAs has been observed in patients with heart and abdominal organ transplants treated with immunosuppressant drugs^56^,^57^. Collectively, although anti-inflammatory therapy has been shown to improve AAA in murine models, an alternative adjunctive therapeutic approach in other pathways is still required. Given the increasing use of HIF-1α inhibitors as an anti-cancer therapy, pharmacological inhibition of aberrantly induced HIF-1α may provide a promising adjunctive medical therapy for patients with small AAA.

Although DFO, digoxin and 2-ME are non-specific HIF-1α modulators, here we have demonstrated the therapeutic potentials of HIF-1a inhibitors as adjunctive medical treatments for AAA. Chemically modified glycosides without cardiac effects have been developed for the anti-cancer therapies^58^. Inhibition of NF-kB/HIF-1 have been shown to decrease IL-17-induced MMP-2 and MMP-9 expression in fibroblasts^59^. Congestive heart failure was independently associated with post-discharge mortality in patients with AAA^60^,^61^,^62^. Digitoxin elicits anti-inflammatory and vasoprotective properties by blocking NF-kB and activating PI-3-kinase/Akt signaling as well as Ca(2+)/Calmodulin-dependent-protein kinase-II in ECs^63^. The dosage used in mice (1–2 mg/kg/day) have not been shown to have adverse effects on the cardiovascular physiology of mice and the serum concentration of digoxin in mice treated at the above-mentioned dose was at or below the therapeutic range for humans (0.5–2.0 ng/ml)^24^,^26^,^27^. Thus, we believe that the beneficial effects of the digoxin/digoxin-like compound should be further explored in the management of small AAAs. It is also worth noting that digoxin (40 μg/day) has been shown to protect against experimental AAA by inhibiting Th17/IL-17A related inflammatory responses^34^. 2-ME is an endogenous natural metabolite of estradiol with HIF-1α inhibition effects^64^,^65^. 2-ME has been shown to reduce monocyte adhesion to ECs and prevents the development of atherosclerosis in ApoE^−/−^ mice^66^,^67^. Given that it has been used in several phase II clinical trials for the treatment of prostate cancer, renal cell carcinoma, and metastatic carcinoid tumors;^68^,^69^,^70^ we believe that the clinical implications could be further expanded in the management of AAA.

### Limitations

Although those HIF-1α modulators are not specific; in conjunction with the facts that HIF-1α accumulation in human AAAs in previous studies, we believe that HIF-1α pathway should be further explored as an intervenable target for stabilizing AAA. We aware that the development of AAA are multi-factorial in origin, and the roles of HIF-1α in the progression and initiation of AAA should be further explored in VSMC as well other inflammatory cells. Since global HIF-1α knockout is lethal, to further elucidate the roles of aberrantly increased HIF-1α in the pathogenesis of AAA, EC or SMC-specific conditional HIF-1α/ApoE triple genetic modified mice, such as tamoxifen-inducible Cre recombinase under target site promotor might be required^42^,^71^. Nonetheless current conditional knock-out inducers, i.e., doxycycline and tamoxifen, had been shown to reduce inflammation and MMPs activations; therefore using those inducers might interfere with further AAA experiments^72^,^73^.

## Conclusion

HIF-1α is pivotal for the development of AAA. Several aneurysmal prone factors cause up-regulation of MMP-2 and MMP-9 through aberrantly induced HIF-1α. Medications that potentiate HIF-1α should be used with caution in patients with AAA. Pharmacological HIF-1α inhibition could attenuate AngII-induced AAA. Our study provides a rationale for using HIF-1α inhibitors as an adjunctive medical therapy in addition to current cardiovascular risk-reducing regimens.

## Additional Information

**How to cite this article**: Tsai, S.-H. *et al*. Inhibition of hypoxia inducible factor-1α attenuates abdominal aortic aneurysm progression through the down-regulation of matrix metalloproteinases. *Sci. Rep.* **6**, 28612; doi: 10.1038/srep28612 (2016).

## Supplementary Material

Supplementary Information
