# Supplementary material for: Inhibition of hypoxia inducible factor-1α attenuates abdominal aortic aneurysm progression through the down-regulation of matrix metalloproteinases
Source: Sci Rep. 2016 Jul 1;6:28612. doi: 10.1038/srep28612 (PMC4929442; doi:10.1038/srep28612)
Supplement: Supplementary Information [file srep28612-s1.pdf]

Inhibition of hypoxia inducible factor-1α attenuates abdominal aortic aneurysm progression through the down-regulation of matrix metalloproteinases

Shih-Hung Tsai[1], Po-Hsun Huang[2,3,4*], Yu-Juei Hsu[5], Yi-Jen Peng[6], Chien-Hsing Lee[7], Jen-Chun Wang[1,2], Jaw-Wen Chen[3,4,8,9], Shing-Jong Lin[2,3,4,9*]

[1] Department of Emergency Medicine, Tri-Service General Hospital, National Defense Medical Center, Taipei, Taiwan,

[2] Institute of Clinical Medicine, National Yang-Ming University, Taipei, Taiwan,

[3] Division of Cardiology, Department of Internal Medicine, Taipei Veterans General Hospital, Taipei, Taiwan,

[4] Cardiovascular Research Center, National Yang-Ming University, Taipei, Taiwan,

[5] Division of Nephrology, Department of medicine, Tri-Service General Hospital, National Defense Medical Center, Taipei, Taiwan,

[6] Department of Pathology, Tri-Service General Hospital, National Defense Medical Center, Taipei, Taiwan,

[7] Division of Endocrinology, Department of medicine, Tri-Service General Hospital, National Defense Medical Center, Taipei, Taiwan,

[8] Institute and Department of Pharmacology, National Yang-Ming University, Taipei, Taiwan,

[9] Department of Medical Research and Education, Taipei Veterans General Hospital, Taipei, Taiwan,

Running title: HIF-1α and abdominal aortic aneurysm

*Corresponding authors:

Dr. Po-Hsun Huang & Professor Shing-Jong Lin

Division of Cardiology, Department of Medicine,

Taipei Veterans General Hospital,

No. 201, Sec. 2, Shih-Pai Road, Taipei, Taiwan.

Tel: + 886-2-2875-7434;

Fax: + 886-2-2875-7435;

E-mail: huangbs@vghtpe.gov.tw & sjlin@vghtpe.gov.tw

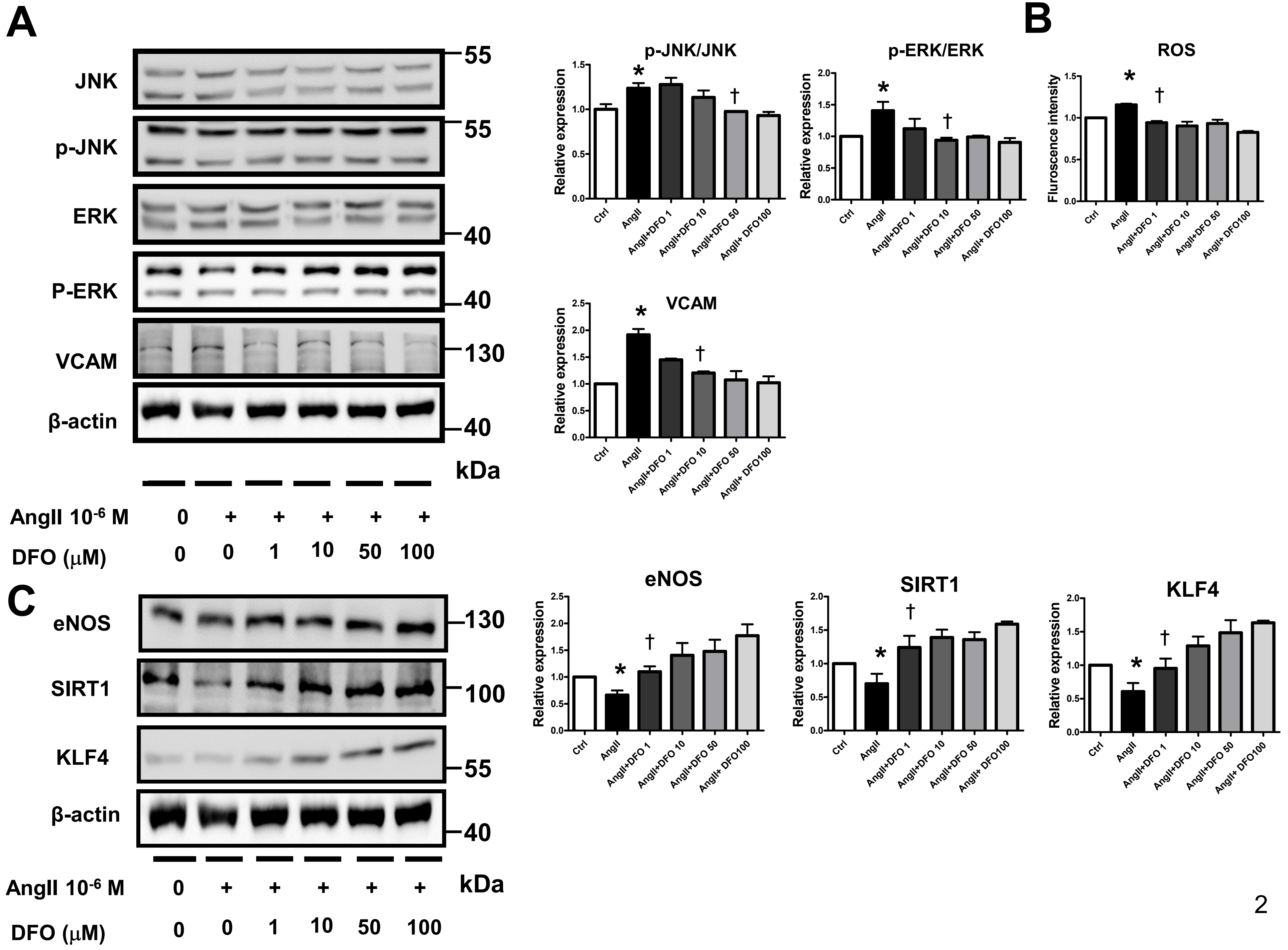
A
JNK
p-JNK
ERK
P-ERK
VCAM
β-actin
55
55
40
40
130
40
kDa
AngII 10-6 M 0 + + + + +
DFO (μM) 0 0 1 10 50 100
B
p-JNK/JNK
p-ERK/ERK
ROS
VCAM
Relative expression
Fluroscence intensity
Ctrl
AngII
AngII+DFO 1
AngII+DFO 10
AngII+DFO 50
AngII+ DFO100
C
eNOS
SIRT1
KLF4
β-actin
130
100
55
40
kDa
AngII 10-6 M 0 + + + + +
DFO (μM) 0 0 1 10 50 100
eNOS
SIRT1
KLF4

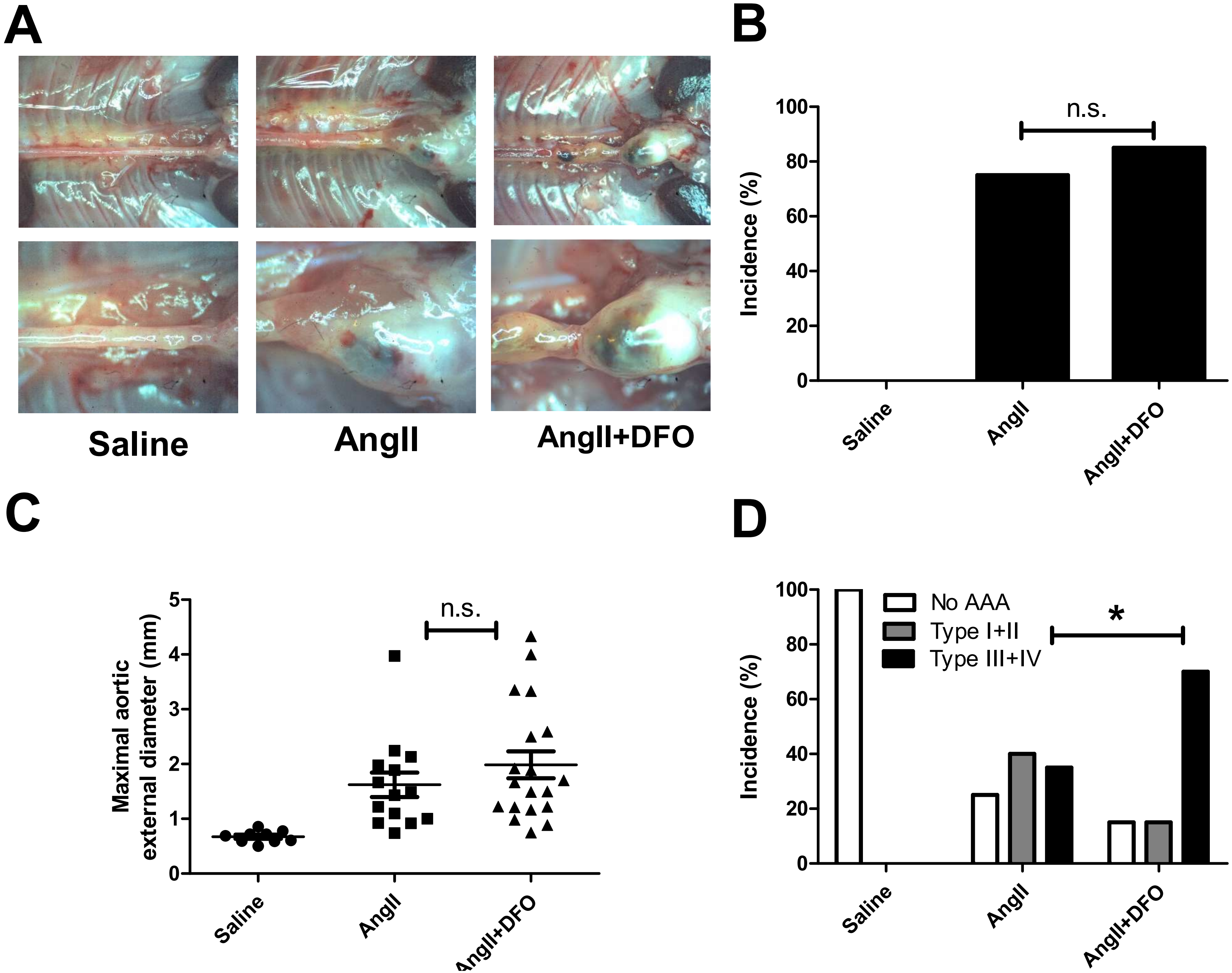

A
B
C
D
Saline
AngII
AngII+DFO
Incidence (%)
n.s.
Maximal aortic external diameter (mm)
No AAA
Type I+II
Type III+IV
*

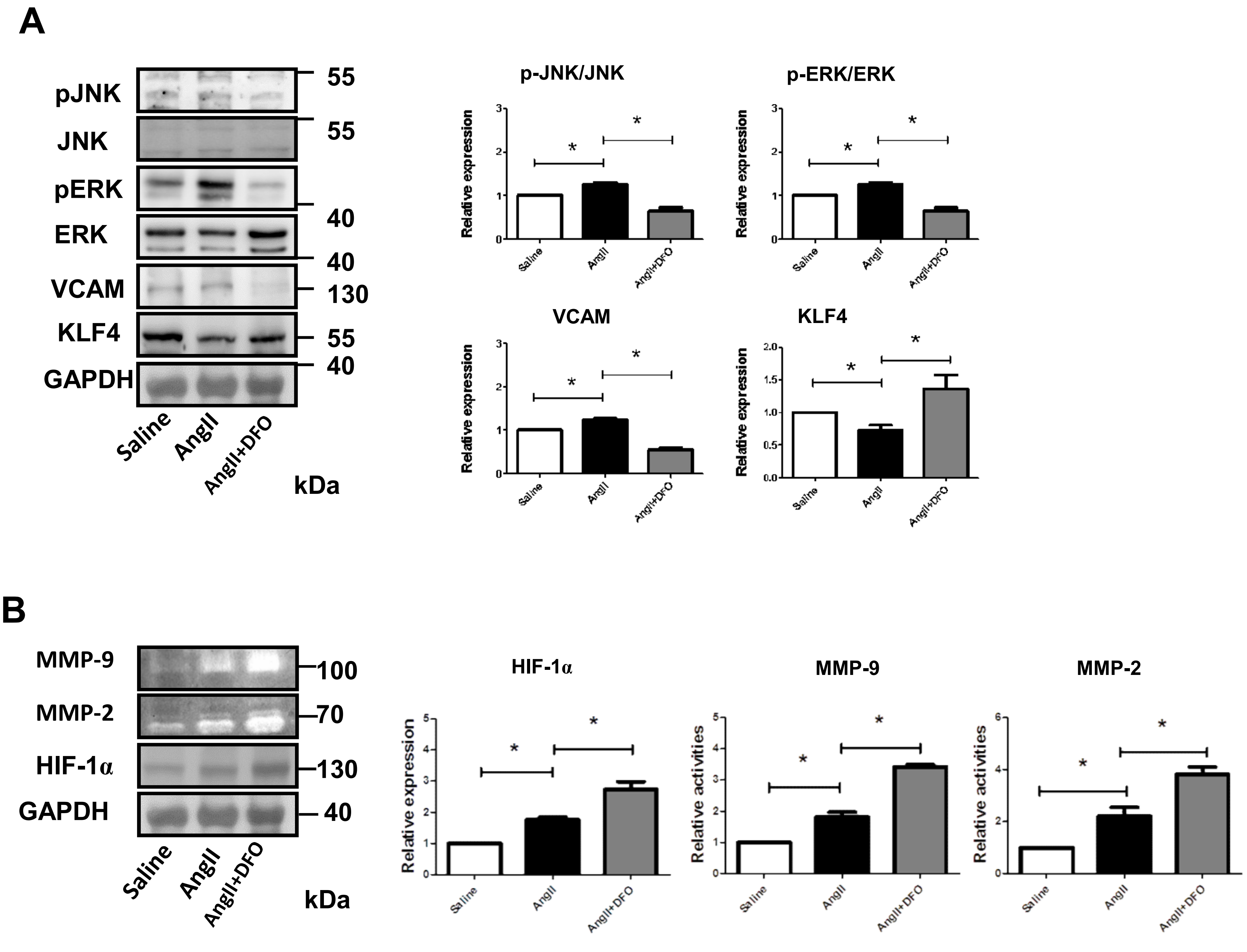
A
pJNK
JNK
pERK
ERK
VCAM
KLF4
GAPDH
55
55
40
40
130
55
40
Saline
AngII
AngII+DFO
kDa
p-JNK/JNK
p-ERK/ERK
VCAM
KLF4
Relative expression
*
B
MMP-9
MMP-2
HIF-1α
GAPDH
100
70
130
40
Saline
AngII
AngII+DFO
kDa
HIF-1α
MMP-9
MMP-2
Relative expression
Relative activities
*

Hypothesis

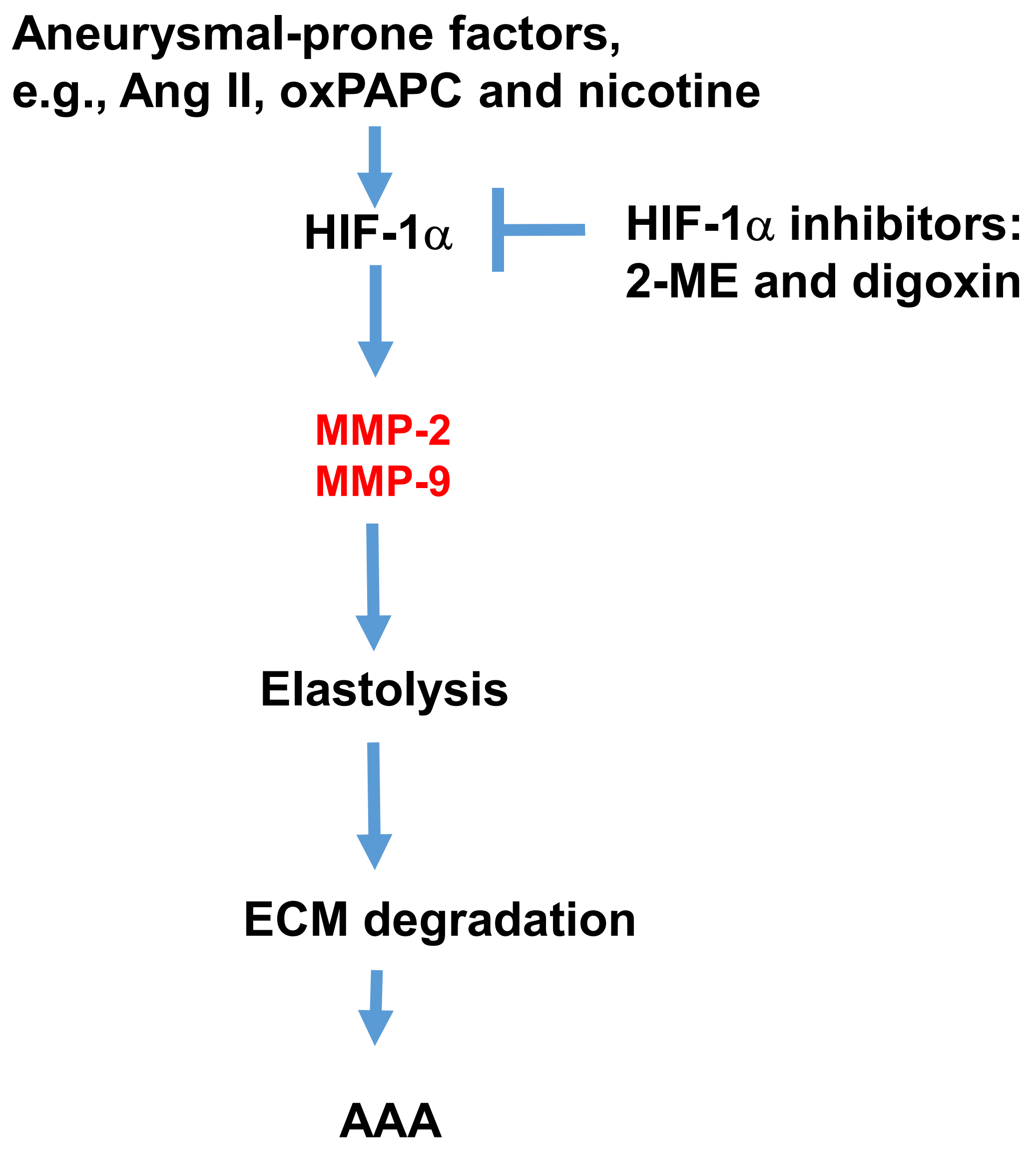
Aneurysmal-prone factors,
e.g., Ang II, oxPAPC and nicotine
HIF-1α
HIF-1α inhibitors:
2-ME and digoxin
MMP-2
MMP-9
Elastolysis
ECM degradation
AAA

Supplementary figures legends

Supplementary figure 1. Deferoxamine attenuates AngII-induced endothelial cell activation and dysfunction *in vitro*

DFO attenuated AngII-induced JNK, ERK1/2 activations and ROS production. In addition, DFO dose-dependently attenuated AngII-induced down-regulation of the athero-protective factors eNOS, SIRT1 and KLR4. The gels have been run under the same experimental conditions. n=4-5**.**

Supplementary figure 2. Deferoxamine does not protect against AngII-induced AAA.

Despite the promising *in vitro* effects, **A,** DFO unexpectedly had the trend toward increasing the incidence of AngII-induced AAA (0% vs. 75% vs. 85%, p=0.69) and **B,** the external diameter of the aorta (0.67±0.11 mm vs. 1.62±0.83 mm vs. 1.98±1.07 mm, p-values: saline vs. AngII, $p< 0.05$; Saline vs. AngII+DFO, $p<0.05$; and AngII vs. AngII+DFO, n.s.). **C.** In addition, we observed a significantly increased incidence of type III (multiple aneurysms) and type IV (rupture) AAA in DFO treated mice (35% vs. 70%, $p<0.05$, n=20).

Supplementary figure 3. Increased HIF-1α expression was associated with increased MMP-2 and MMP-9 activity *in vitro*

A, *In vivo*, DFO could attenuate AngII induced JNK and ERK activation; VCAM overexpression and incease athero-protective KLF4. **B**, To further elucidate how DFO treatment could augment AAA severity, we tested HIF-1α expression in the whole aorta lysate and found that compared with the AngII-treated group, DFO treated mice had significantly higher HIF-1α expression in the aorta lysate and significantly increased MMP-2 and MMP-9 activities **(**n=5). The gels have been run under the same experimental conditions.
